# The moderating effect of cognitive re-evaluation on the relationship between superficial performance and job burnout in psychiatric nurses

**DOI:** 10.3389/fpsyt.2026.1929514

**Published:** 2026-10-14

**Authors:** Shasha Hong, Shuhui Huang, Mianmian Zhu, Aimin Jin, Qihao Zhang, Anmeng Huang, Lu Wang

**Affiliations:** 1 Department of Comprehensive Spirit III, Wenzhou Seventh People’s Hospital, Wenzhou, Zhejiang, China; 2 Department of Ward 502, Wenzhou Seventh People’s Hospital, Wenzhou, Zhejiang, China; 3 Department of Psychosomatic Clinic, Wenzhou Seventh People’s Hospital, Wenzhou, Zhejiang, China; 4 Department of the Third Ward, Wenzhou Seventh People’s Hospital, Wenzhou, Zhejiang, China; 5 Department of Alcohol Dependence Ward, Wenzhou Seventh People’s Hospital, Wenzhou, Zhejiang, China; 6 Department of Ward 301, Wenzhou Seventh People’s Hospital, Wenzhou, Zhejiang, China

**Keywords:** emotion regulation, occupational burnout, psychiatric nurse, regulatory effect, surface acting

## Abstract

**Background:**

Demand for emotional labor is increasing among psychiatric nurses who are now more at risk for job burnout, and emotional labor strategies or emotion regulation styles may be important mechanisms in this process.

**Aim:**

The purpose of this study was to investigate the relationships among emotional labor, emotion regulation, and job burnout in psychiatric nurses, and to examine cognitive reappraisal as a potential moderator of the relationship between surface acting and burnout.

**Methods:**

During January-February 2025, a cross-sectional survey was performed among on-duty nurses in a tertiary psychiatric hospital located in Zhejiang Province, China. Emotional labor, emotion regulation, job burnout, nursing professional values, perceived decent work and innovative capacity were assessed using validated Chinese scales. Descriptive statistics, correlation analyses, multiple linear regression, hierarchical moderation analysis, and simple slope analysis were performed. Regression assumptions, multicollinearity, and common method bias were additionally assessed.

**Results:**

Of 245 returned questionnaires, 241 were included in the final analysis. After adjustment for demographic and work-related factors, surface acting was positively associated with job burnout (β = 0.416, 95% CI: 0.249–0.582, p < 0.001), whereas cognitive reappraisal was inversely associated with burnout (β = −0.419, 95% CI: −0.560 to −0.278, p < 0.001). The surface acting × cognitive reappraisal interaction was statistically significant but small (β = −0.137, 95% CI: −0.238 to −0.036, ΔR² = 0.019, f² = 0.032, p = 0.008).

**Conclusion:**

Higher surface acting was associated with higher job burnout, whereas higher cognitive reappraisal was associated with lower burnout. Cognitive reappraisal also showed a small moderating association, with the positive association between surface acting and burnout becoming weaker at higher levels of reappraisal. Longitudinal studies are needed to clarify the temporal direction and practical relevance of these associations.

## 1 Introduction

Nurse burnout is now recognized as a major problem in nursing worldwide and has implications for the tension rising between nurses and patients, which in turn increases complexity of health-care needs. Burnout is usually characterized by emotional depletion, depersonalization, and a diminished sense of personal accomplishment as a result of prolonged work stress (1–3). There is a large body of research supporting that increased nurse burnout may be linked to lower quality care, more adverse events, decreased patient satisfaction, greater nurse job turnover, and poorer quality of life for nurses (1–5). These undesirable consequences make nurses’ physical and psychological well-being vulnerable and challenge the capacity and safety of health-care systems (2, 4, 5).

Psychiatric nurses are vulnerable to occupational burnout. They are more likely than nurses in most other fields to experience violence or aggression, to have to deal with self-harm incidents, and to care for individuals with chronic, treatment-resistant mental illness, all the while needing to have stable therapeutic relationships over extended periods (6–8). International research indicates that psychiatric nurses experience high levels of emotional exhaustion, depression, anxiety, and poor quality of life, in part related to heavy workloads, role ambiguity, and stigma associated with mental health services (6–8). These results suggest an urgent requirement to identify a modifiable psychological mechanism that may either exacerbate or buffer burnout among psychiatric nurses.

Emotional labor is a core psychological demand in nursing work, referring to the process by which employees manage their emotions to meet organizational display rules and patient expectations (9, 10). A commonly used framework differentiates between surface acting and deep acting: surface acting involves faking or suppressing emotions without changing internal feelings, essentially putting on a mask, whereas deep acting involves actively modifying internal appraisals so that genuinely felt emotions become congruent with required expressions (9, 10). Studies of health professionals have shown that surface acting is closely and positively related to emotional exhaustion and job burnout, whereas results for deep acting are mixed or even indicate a negative association with burnout (9, 10). This is significant because psychiatric nurses who require high levels of empathy and emotional containment daily may be most at risk from relying on surface acting, such as “faking it”, which may, over time, contribute to higher burnout levels in this population (6, 7, 9, 10).

Emotion regulation refers to the processes by which people affect what emotions they have, when they have them, and how they experience and express these emotions (11). Cognitive re-evaluation, also known as cognitive reappraisal, and expressive suppression are two of the most extensively investigated strategies. Reappraisal entails changing the meaning of an emotionally salient event to modulate its emotional impact prior to a full emotional response being initiated, while suppression involves concealing the behavioral manifestation of pre-experienced emotions (11, 12). An abundance of behavioral and neuroimaging research has shown that reappraisal not only decreases self-reported negative affect and amygdala activation but also increases activation in prefrontal areas involved in cognitive control. In contrast, suppression has been shown to have a reversed effect on these measures, and it is more cognitively demanding, is associated with increased sympathetic activation, and leads to poorer social consequences (12).

Recent findings in healthcare professionals suggest that adaptive emotion regulation strategies, including cognitive reevaluation, are associated with decreased burnout, improved psychological well-being, and greater professional quality of life, while maladaptive strategies, such as pervasive suppression, correlate with depressive symptomatology and secondary traumatic stress (13, 14). In a sample of medical residents and other healthcare providers, more frequent use of cognitive reappraisal was associated with less burnout and a more positive work-life balance, over and above the effects of workload and demographics (13). In mental health care professionals, superior emotion regulation skills, including reappraisal and mindfulness, have been found to correlate with greater compassion satisfaction and reduced burnout (14). These findings suggest that cognitive reappraisal may be a personal psychological resource that helps clinicians persist with high emotional load.

However, relatively few studies have examined how specific emotion regulation strategies interact with emotional labor to influence burnout in nurses, and even fewer have focused on psychiatric nurses (9, 10, 13–15). Recent work has begun to explore whether emotion regulation can buffer the harmful effects of surface acting on burnout, but the available evidence remains limited in terms of sample size, occupational specificity, and the granularity with which emotional labor dimensions and burnout domains are measured (9, 10, 15). In particular, it is still unclear whether cognitive re-evaluation can weaken the positive association between surface acting, or superficial role-playing, and job burnout among psychiatric nurses, who experience intense and sustained emotional demands.

Therefore, the present study focuses on psychiatric nurses in China and aims to address these gaps. Specifically, we first describe the current status of emotional labor, with a focus on surface acting, as well as cognitive re-evaluation and occupational burnout in this population. Second, we examine the direct relationship between surface acting and job burnout. Third, and most crucial, we examine whether cognitive reappraisal moderates the relation between surface acting and burnout, such that higher cognitive reappraisal buffers the negative effect of surface acting on burnout. Elucidating this moderating mechanism could provide theoretical support and empirical evidence for the design and evaluation of focused emotion regulation enhancement interventions to best protect the mental health and professional sustainability of psychiatric nurses (13–15).

## 2 Materials and methods

### 2.1 Study subjects

This cross-sectional study recruited on-duty psychiatric nurses from Wenzhou Seventh People’s Hospital between 20 January and 1 February 2025 using a hospital-wide convenience sampling approach across the participating psychiatric wards. Inclusion criteria were: (1) possession of a valid National Registered Nurse qualification; (2) currently working in clinical psychiatric nursing; (3) provision of written informed consent; and (4) no self-reported history of mental illness. Exclusion criteria were: (1) intern or trainee nurses and (2) nurses not currently on active duty.

Based on the rule of at least 10–20 participants per independent variable (n = 10) and allowing for approximately 20% invalid questionnaires, we estimated a minimum required sample size of 240. Because moderation analysis was a primary objective, a sensitivity power analysis was conducted. With N = 241, a two-sided α of 0.05, and 80% statistical power, the minimum detectable incremental effect for a single interaction term was approximately f^2^ = 0.033. A total of 245 questionnaires were distributed and returned. After quality control procedures described in Section 2.2.5, four questionnaires were excluded because three had completion times under 100 seconds and one had an implausible value for years of work. The final analytic sample comprised 241 nurses, corresponding to an effective response rate of 98.4%.

### 2.2 Survey tools

#### 2.2.1 General situation survey form

Demographic and work-related characteristics were collected using a self-designed questionnaire. The instrument comprised two modules: (1) a demographic module covering gender, age, highest educational level, professional title, and years of work experience; and (2) a health behavior module assessing night-shift frequency, exercise frequency, and dietary habits.

#### 2.2.2 Emotional labor

Emotional labor was assessed using the Emotional Labor Scale (ELS) developed by Diefendorff et al (16), which includes three dimensions: (1) surface acting, reflecting the discrepancy between outward emotional display and inner experience; (2) deep acting, describing efforts to align internal feelings with required emotional expressions through cognitive adjustment; and (3) expression of naturally felt emotions. This study used the Chinese revised version of the ELS developed by Bai et al (17), where the Cronbach’s α coefficient for the total scale was 0.89 and 0.91 for the surface acting subscale. In the current research, internal consistency of the total scale and subscales also reached acceptable levels (α > 0.80).

#### 2.2.3 Occupational burnout

Occupational burnout was measured using the Chinese version of the Maslach Burnout Inventory–General Survey (MBI-GS) (18), which was jointly developed by Maslach and Jackson, comprises three dimensions: emotional exhaustion, cynicism or depersonalization, and reduced personal accomplishment, with a total of 15 items rated on a 7-point Likert scale (0 = “never” to 6 = “every day”). Higher scores indicate higher levels of burnout. In this study, the Cronbach’s α coefficient for the total scale was 0.89, and the α coefficients for emotional exhaustion, cynicism, and reduced personal accomplishment were 0.96, 0.94, and 0.96, respectively.

#### 2.2.4 Emotional regulation

Emotion regulation was assessed using the Emotion Regulation Questionnaire (ERQ), originally developed by Gross and John and subsequently revised into a Chinese version by Wang et al. (11, 19) The ERQ consists of 10 items rated on a 7-point Likert scale, with higher scores indicating more frequent use of the corresponding strategy. It comprises two dimensions: cognitive reappraisal (6 items) and expressive suppression (4 items). In the present study, the internal consistency coefficients for cognitive reappraisal and expressive suppression were 0.93 and 0.90, respectively.

#### 2.2.5 Data collection and quality control

Completion time was used as an indicator of potentially insufficient-effort responding (20). The complete questionnaire contained 96 scale items in addition to eight demographic and work-related questions. Completion in less than 100 seconds would therefore require approximately one second or less per item on average, which was considered implausibly rapid for meaningful reading and responding. Accordingly, responses completed in less than 100 seconds were excluded. Records containing clearly implausible demographic values were also reviewed during data cleaning. Three responses were excluded because of completion times below 100 seconds and one because of an implausible value for years of work, resulting in a final analytic sample of 241 nurses.

### 2.3 Statistical methods

All analyses were carried out using SPSS version 26.0 (IBM Corp., Armonk, NY, USA). We deemed p-values < 0.05 (two-sided) significant.

#### 2.3.1 Descriptive analyses and normality assessment

The characteristics of the sample were first summarized using descriptive statistics. Categorical variables were expressed as frequency and percentage [n (%)], while continuous variables were expressed as mean ± standard deviation (M ± SD). Distributional characteristics were assessed using skewness and kurtosis together with the Shapiro–Wilk and Kolmogorov–Smirnov tests. Because formal normality tests can be sensitive to small departures from normality in moderate-sized samples, distributional shape and regression residual diagnostics were also considered. Pearson correlation coefficients were used for the primary bivariate analyses, and Spearman rank correlations were conducted as a sensitivity analysis to assess the robustness of the observed associations (21, 22).

#### 2.3.2 Multiple regression analyses of job burnout

In order to further explore predictors of job burnout, a series of multiple linear regression analyses was run with the overall burnout score as the outcome variable. In Model 1 as the baseline model, demographic and work-related factors (gender, age, level of education, working years, professional title, frequency of night-shift, frequency of exercise, and eating habits) were regarded as covariates. Model 2 also included the emotional labor factors (surface acting, deep acting, and expression of naturally felt emotions), and the emotion regulation strategies cognitive reappraisal and expressive suppression.

Categorical covariates with an inherent ordinal structure were entered as ordinal variables. Dietary habits were treated as a nominal categorical variable and dummy coded, with “regular three meals on time” as the reference category (**Table 1**). Meanwhile, the multicollinearity was assessed using variance inflation factors (VIFs) and tolerance values. Regression assumptions were examined before model interpretation. Linearity and homoscedasticity were assessed using residual-versus-fitted plots, residual normality was examined using normal probability plots and formal tests, independence of errors was evaluated using the Durbin–Watson statistic, and potentially influential observations were assessed using studentized residuals and Cook’s distance. The Breusch–Pagan and White tests were additionally used to assess heteroscedasticity.

**Table 1:** Sociodemographic and work-related characteristics of psychiatric nurses (categorical part).

Variable	Category	n (%)
Gender		
	Male	18 (7.5%)
	Female	223 (92.5%)
Educational level		
	High school/vocational and below	4 (1.7%)
	Junior college	40 (16.6%)
	Bachelor’s degree or above	197 (81.7%)
Professional title		
	Nurse	54 (22.4%)
	Primary nurse	143 (59.3%)
	Nurse-in-charge	37 (15.4%)
	Associate chief nurse	5 (2.1%)
	Chief nurse	2 (0.8%)
Night shift frequency (per month)		
	< 1 time	31 (12.9%)
	1–4 times	199 (82.6%)
	5–8 times	9 (3.7%)
	> 8 times	2 (0.8%)
Exercise frequency		
	Never	42 (17.4%)
	Occasionally	163 (67.6%)
	1–2 times per week	26 (10.8%)
	≥ 3 times per week	10 (4.1%)
Dietary habits		
	Regular three meals on time	102 (42.3%)
	Occasionally irregular	82 (34.0%)
	Often irregular	30 (12.4%)
	Mainly takeout/fast food	27 (11.2%)

#### 2.3.3 Moderation and simple slope analyses

Hierarchical regression analysis was conducted to examine the moderating role of cognitive reappraisal between surface acting and job burnout. Model 1 included the demographic and work-related covariates specified above; Model 2 added surface acting, cognitive reappraisal, and expressive suppression; and Model 3 entered the interaction term of surface acting and cognitive reappraisal. Job burnout, surface acting, cognitive reappraisal, and expressive suppression were z-standardized before analysis. The interaction term was created by multiplying the standardized surface acting and cognitive reappraisal variables.

Significant interaction effects were further probed using simple slope analyses. The conditional effects of surface acting on job burnout were estimated at low (−1 SD), mean (0 SD), and high (+1 SD) levels of cognitive reappraisal, while controlling for demographic and work-related covariates. The results were summarized in a simple slope table and illustrated graphically using a simple slope plot.

#### 2.3.4 Common method bias and sensitivity analyses

Because the principal study variables were assessed using self-report questionnaires collected at a single time point, potential common method bias was examined using Harman’s single-factor test (23). All questionnaire items were entered simultaneously into an unrotated exploratory factor analysis, and the proportion of variance explained by the first factor was examined. A first-factor variance below 40% was considered to indicate that no dominant single-factor method effect was evident. In addition, Spearman correlations were used as a sensitivity analysis. Where regression diagnostics suggested potential heteroscedasticity, robust and bootstrap-based inference was examined to assess the stability of the moderation result.

## 3 Results

### 3.1 General information of psychiatric nurses

After data screening, the final sample consisted of 241 nurses, where females were predominantly distributed, and most participants were young to middle-aged with moderate work experience. The majority held a bachelor’s degree or above and were at the junior or intermediate professional level. Night shifts were common, and a notable proportion of nurses reported limited physical exercise and irregular dietary habits. Formal normality tests indicated departures from exact normality for several continuous variables; however, skewness and kurtosis values remained within the prespecified acceptable ranges. The robustness of the bivariate findings was therefore additionally examined using Spearman rank correlations. Detailed scale characteristics are presented in **Table 2**, and demographic and work-related characteristics are presented in **Table 1**.

**Table 2:** Sociodemographic and work-related characteristics of psychiatric nurses (continuous part).

Variable	Category	M ± SD	Skewness	Kurtosis
Age (years)		30.85 ± 6.22	1.39	2.79
Working life (years)*		8.48 ± 5.41	1.66	3.98
Emotional labor				
	Surface acting	21.89 ± 5.50	-0.07	0.51
	Deep acting	13.88 ± 2.85	-0.18	1.07
	Natural expression	10.60 ± 1.85	0.28	1.09
Emotion regulation				
	Cognitive reappraisal	30.49 ± 6.46	0.32	-0.51
	Expressive suppression	17.07 ± 4.93	0.24	0.38
Job burnout (total score)		42.30 ± 20.07	-0.47	-0.48
Nursing professional values	Total score	101.95 ± 19.95	-0.19	-0.82
Perceived decent work	Total score	43.72± 6.96	0.46	0.17
Innovative capacity	Total score	33.62 ± 6.91	0.62	0.08

Data are presented as mean ± standard deviation (SD). Skewness and kurtosis were used to assess normality.

### 3.2 Descriptive statistics and correlations among key psychological variables

Descriptive statistics and Pearson correlations for emotional labor, emotion regulation, job burnout, and work-related psychological variables are summarized in **Table 3**. Correlation analyses indicated that higher surface acting was associated with higher overall job burnout and with greater emotional exhaustion and depersonalization, whereas its association with reduced personal accomplishment was small and negative. Deep acting and natural expression were weakly and negatively associated with burnout. Cognitive reappraisal showed a clear inverse association with burnout and was positively associated with professional values, perceived decent work, and innovative capacity. Expressive suppression showed a weak positive association with burnout. Perceived decent work, professional values, and innovative capacity were consistently associated with lower burnout.

**Table 3:** Descriptive statistics and Pearson correlations among emotional labor, emotion regulation, burnout, and work-related psychological variables.

Variable	M	SD	Skewness	Kurtosis	1	2	3	4	5	6	7	8	9	10	11	12
1. Surface acting	21.89	5.50	-0.07	0.51	—											
2. Deep acting	13.88	2.85	-0.18	1.07	0.64	—										
3. Natural expression	10.60	1.85	0.28	1.09	0.54	0.71	—									
4. Cognitive reappraisal	30.49	6.46	0.32	-0.51	0.30	0.57	0.57	—								
5. Expressive suppression	17.07	4.93	0.24	0.38	0.66	0.49	0.42	0.44	—							
6. Job burnout (total)	42.30	20.07	-0.47	-0.48	0.26	-0.13	-0.21	-0.41	0.13	—						
7. Emotional exhaustion	16.29	6.52	0.59	0.31	0.37	0.11	0.01	-0.12	0.20	0.78	—					
8. Depersonalization	11.35	5.75	0.79	0.48	0.37	0.03	-0.01	-0.14	0.25	0.79	0.85	—				
9. Reduced personal accomplishment	14.66	7.80	-0.09	-0.63	-0.09	-0.32	-0.36	-0.53	-0.10	0.60	0.00	0.05	—			
10. Nursing professional values	101.95	19.95	-0.19	-0.82	0.18	0.42	0.37	0.47	0.23	-0.34	-0.09	-0.15	-0.41	—		
11. Perceived decent work	43.72	6.96	0.46	0.17	-0.09	0.22	0.31	0.43	0.15	-0.63	-0.52	-0.49	-0.36	0.45	—	
12. Innovative capacity	33.62	6.91	0.62	0.08	0.13	0.39	0.47	0.52	0.24	-0.34	-0.05	-0.01	-0.54	0.42	0.38	—

M, mean; SD, standard deviation. Values are Pearson correlation coefficients. Coefficients with |r| ≥ 0.13 were statistically significant at p < 0.05.

Spearman sensitivity analyses produced associations that were similar in direction and magnitude to the primary Pearson correlations. For example, the surface acting–burnout association was r = 0.255 using Pearson correlation and ρ = 0.281 using Spearman correlation, whereas the cognitive reappraisal–burnout association was r = −0.414 and ρ = −0.389, respectively. Harman’s single-factor analysis extracted 15 factors with eigenvalues greater than 1; the first unrotated factor accounted for 31.78% of the total variance, below the prespecified 40% threshold.

### 3.3 Multiple regression analysis of emotional labor, emotion regulation, and job burnout

The results of multiple linear regression are presented in **Table 4**. Model 1, which included demographic and work-related covariates, explained 9.4% of the variance in burnout (R² = 0.094, adjusted R² = 0.055, F = 2.392, p = 0.010). After emotional labor and emotion regulation variables were added, Model 2 explained 41.6% of the variance (R² = 0.416, adjusted R² = 0.377, F = 10.678, p < 0.001; ΔR² = 0.322).

**Table 4:** Multiple linear regression of job burnout on emotional labor and emotion regulation after adjustment for demographic and work-related variables.

Variable	Model 1 β (95% CI)	t	p	Model 2 β (95% CI)	t	p	VIF	Tolerance
Demographic and work-related variables								
Age (years)	0.090 (−0.256 to 0.435)	0.511	0.61	−0.028 (−0.312 to 0.257)	−0.193	0.847	8.029	0.125
Years of work	−0.329 (−0.661 to 0.004)	−1.950	0.053	−0.160 (−0.437 to 0.118)	−1.136	0.257	7.633	0.131
Gender	−0.119 (−0.245 to 0.008)	−1.846	0.066	−0.112 (−0.215 to −0.008)	−2.128	0.034	1.064	0.94
Education level	0.106 (−0.032 to 0.243)	1.515	0.131	0.051 (−0.062 to 0.164)	0.891	0.374	1.263	0.792
Professional title	0.240 (0.027 to 0.453)	2.221	0.027	0.205 (0.030 to 0.381)	2.304	0.022	3.056	0.327
Night-shift frequency	0.040 (−0.089 to 0.169)	0.609	0.543	0.010 (−0.098 to 0.118)	0.185	0.853	1.16	0.862
Exercise frequency	−0.002 (−0.130 to 0.126)	−0.035	0.972	−0.001 (−0.106 to 0.104)	−0.020	0.984	1.087	0.92
Dietary habits — reference: regular three meals on time								
Occasionally irregular	0.146 (0.004 to 0.288)	2.021	0.045	0.062 (−0.055 to 0.178)	1.041	0.299	1.349	0.741
Often irregular	0.054 (−0.086 to 0.193)	0.758	0.449	0.023 (−0.092 to 0.139)	0.395	0.693	1.319	0.758
Mainly takeout/fast food	0.101 (−0.041 to 0.242)	1.403	0.162	0.084 (−0.033 to 0.200)	1.42	0.157	1.344	0.744
Emotional labor and emotion regulation								
Surface acting	—	—	—	0.416 (0.249 to 0.582)	4.929	<0.001	2.739	0.365
Deep acting	—	—	—	−0.145 (−0.317 to 0.027)	−1.660	0.098	2.931	0.341
Natural expression	—	—	—	−0.172 (−0.326 to −0.019)	−2.218	0.028	2.33	0.429
Cognitive reappraisal	—	—	—	−0.419 (−0.560 to −0.278)	−5.863	<0.001	1.969	0.508
Expressive suppression	—	—	—	0.174 (0.027 to 0.322)	2.332	0.021	2.151	0.465

β values are standardized regression coefficients. Dietary habits were dummy coded with “regular three meals on time” as the reference category. VIF and tolerance values are reported for Model 2.

In the fully adjusted model, surface acting was positively associated with burnout (β = 0.416, 95% CI: 0.249–0.582, p < 0.001), whereas cognitive reappraisal was inversely associated with burnout (β = −0.419, 95% CI: −0.560 to −0.278, p < 0.001). Natural expression was also inversely associated with burnout (β = −0.172, 95% CI: −0.326 to −0.019, p = 0.028), while expressive suppression showed a positive association (β = 0.174, 95% CI: 0.027–0.322, p = 0.021). Deep acting was not independently associated with burnout after adjustment (β = −0.145, 95% CI: −0.317 to 0.027, p = 0.098).

The highest VIF was observed for age (8.029), followed by years of work (7.633). VIFs for the principal emotional labor and emotion regulation variables ranged from 1.969 to 2.931. Sensitivity analyses excluding either age or years of work produced materially unchanged estimates for the principal psychological predictors.

### 3.4 Moderating effect of cognitive reappraisal and simple slope analysis

Hierarchical moderation analysis is presented in **Table 5**. The surface acting × cognitive reappraisal interaction was statistically significant (β = −0.137, 95% CI: −0.238 to −0.036, p = 0.008). Addition of the interaction term increased the explained variance by 1.9% (ΔR² = 0.019), corresponding to a small incremental effect (Cohen’s f² = 0.032).

**Table 5:** Hierarchical regression models testing the moderating effect of cognitive reappraisal on the association between surface acting and job burnout.

Predictor	Model 1 β	Model 2 β	Model 3 β (95% CI)
Demographic and work-related variables			
Age (years)	0.09	−0.045	0.024
Years of work	−0.329	−0.114	−0.151
Gender	−0.119	−0.122*	−0.120*
Educational level	0.106	0.045	0.037
Professional title	0.240*	0.188*	0.187*
Night-shift frequency	0.04	−0.005	−0.018
Exercise frequency	−0.002	0.011	0.016
Dietary habits — reference: regular three meals on time			
Occasionally irregular	0.146*	0.073	0.075
Often irregular	0.054	0.024	0.034
Mainly takeout/fast food	0.101	0.078	0.089
Emotional labor and emotion regulation			
Surface acting (standardized)	—	0.266***	0.331 (0.180 to 0.481)*
Cognitive reappraisal (standardized)	—	−0.566***	−0.594 (−0.711 to −0.477)*
Expressive suppression (standardized)	—	0.199**	0.259 (0.104 to 0.413)
Interaction term			
Surface acting × cognitive reappraisal	—	—	−0.137 (−0.238 to −0.036)

**β values are standardized regression coefficients. Dietary habits were dummy coded with “regular three meals on time” as the reference category. Job burnout, surface acting, cognitive reappraisal, and expressive suppression were z-standardized before analysis. Demographic and work-related covariates were adjusted in all models. Model 1: R² = 0.094, adjusted R^2^ = 0.055. Model 2: R^2^ = 0.383, adjusted R^2^ = 0.348. Model 3: R^2^ = 0.402, adjusted R^2^ = 0.365; ΔR^2^ = 0.019, F-change = 7.198, p = 0.008. The incremental interaction effect was small (Cohen’s f² = 0.032). *p < 0.05; **p < 0.01; ***p < 0.001.

Simple slope analyses are presented in **Table 6** and **Figure 1**. The positive association between surface acting and burnout was strongest at low cognitive reappraisal (β = 0.47, 95% CI: 0.26–0.67, p < 0.001), weaker at the mean level (β = 0.33, 95% CI: 0.18–0.48, p < 0.001), and weakest but still statistically significant at high cognitive reappraisal (β = 0.19, 95% CI: 0.04–0.35, p = 0.013).

**Table 6:** Simple slope analysis of the association between surface acting and job burnout at different levels of cognitive reappraisal.

Level of cognitive reappraisal	Slope of surface acting (β)	SE	95% CI for β	t	p
Low (−1 SD)	0.47	0.1	0.26 to 0.67	4.48	<0.001
Mean (0 SD)	0.33	0.08	0.18 to 0.48	4.32	<0.001
High (+1 SD)	0.19	0.08	0.04 to 0.35	2.5	0.013

Job burnout, surface acting, and cognitive reappraisal were standardized (z-scores). Simple slopes were estimated at low (−1 SD), mean (0 SD), and high (+1 SD) levels of cognitive reappraisal, controlling for demographic and work-related covariates.

**Figure 1:**
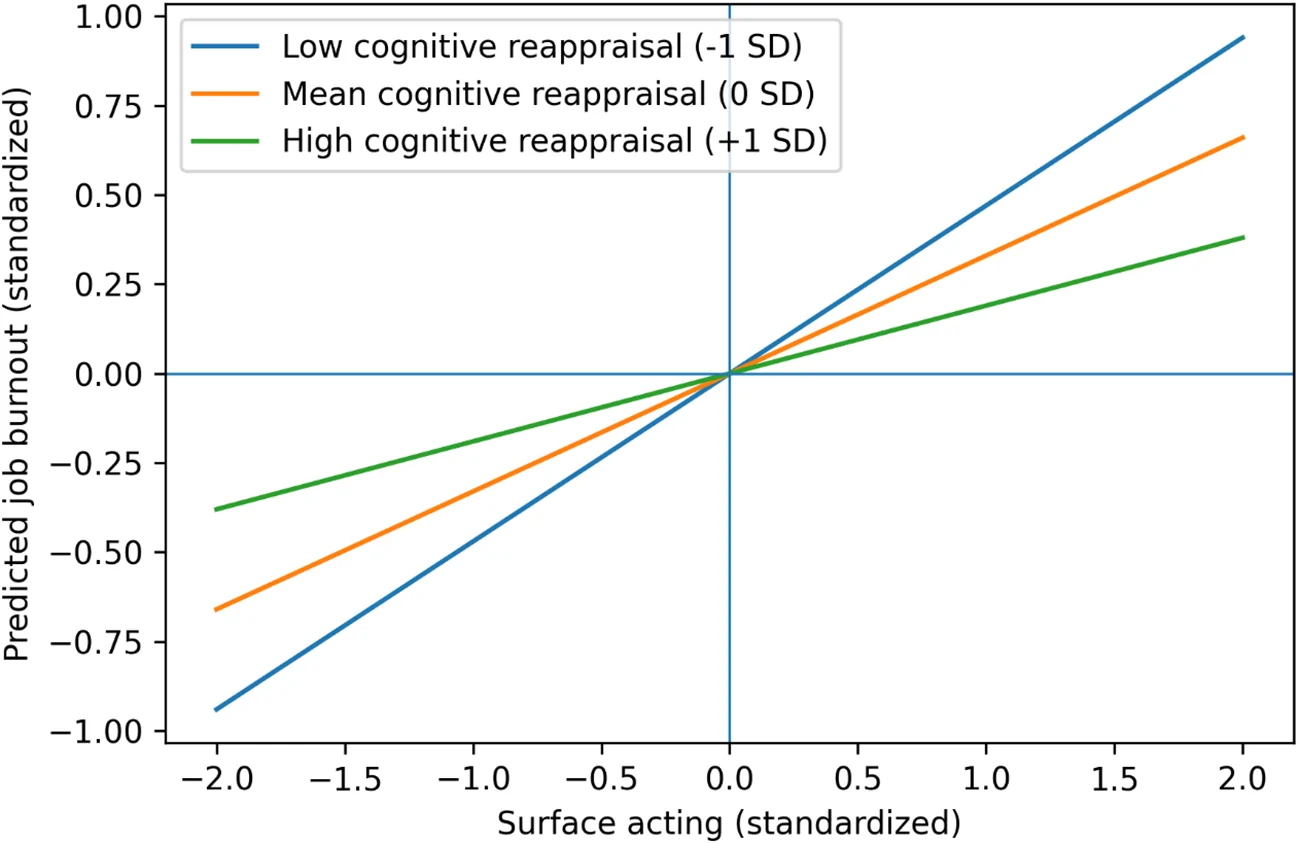
Simple slopes of surface acting predicting job burnout at different levels of cognitive reappraisal. Job burnout, surface acting, and cognitive reappraisal were standardized (z-scores). Lines represent the simple slopes of surface acting at low (−1 SD), mean (0 SD), and high (+1 SD) levels of cognitive reappraisal, controlling for demographic and work-related covariates.

## 4 Discussion

This study found that psychiatric nurses in a large Chinese psychiatric hospital experience a notable level of job burnout, with a clear gradient pattern in emotional exhaustion, depersonalization and reduced personal accomplishment. This aligns with national and international evidence that mental health nurses have high burnout levels and that emotional demands are important drivers of this problem (24, 25). Our findings also indicated that emotional labor and emotion regulation strategies account for more variance in burnout than demographic or work schedule factors.

Surface acting was consistently associated with a higher burnout. Nurses who frequently relied on superficial emotional displays were more exhausted, more detached and reported poorer professional fulfilment. This is consistent with recent work among nurses and other health professionals, where surface acting has been linked to increased emotional exhaustion, stronger job stress, lower work engagement and a greater intention to leave (26–28). Conceptual models suggest that surface acting may contribute to chronic emotional dissonance and sustained depletion of personal resources (26, 29). For psychiatric nurses, who must manage aggression, self-harm risk and complex family interactions, repeated suppression of genuine fear or frustration in favor of required emotional displays may gradually erode energy and empathy (24, 30).

In contrast, cognitive reappraisal showed a clear and consistent association with lower burnout and with more positive work related attitudes. Nurses who more often reframed difficult situations reported less burnout and higher professional values, perceived their work as more decent and showed stronger innovative capacity. This pattern is in line with the process model of emotion regulation and with studies of health professionals in different settings, which have shown that adaptive strategies such as cognitive reappraisal are associated with less mental distress, whereas expressive suppression is related to higher depression, anxiety and burnout (31–33). Research in medical staff and residents has further demonstrated that emotion regulation difficulties predict higher burnout over time and that cognitive reappraisal can partly mediate the impact of stress on exhaustion (31, 34–36). Our results extend this evidence to psychiatric nurses in China and show that the protective role of cognitive reappraisal remains significant after accounting for expressive suppression, emotional labor and background factors.

A key contribution of this study is that it suggests that cognitive reappraisal moderates the relationship between surface acting and burnout. The positive association between surface acting and burnout was strongest when cognitive reappraisal was low and weaker when cognitive reappraisal was average or high. This graded pattern indicates that cognitive reappraisal acts as a buffer but does not completely neutralize the strain generated by surface acting. Similar findings have been reported in a recent study of Chinese psychiatric nurses in which cognitive reappraisal and other emotion regulation strategies altered the strength of associations between emotional labor and burnout (15). Other work in healthcare workers and trainees also points to emotion regulation as a key mediator or moderator that shapes how workload, mistreatment and workplace violence translate into burnout or resilience (33, 36, 37).

Our results suggest that psychiatric nurses may hardly avoid surface acting, since patient safety and therapeutic communication sometimes require emotional displays that do not fully match internal states. But nurses who can reframe aggression as a symptom of illness, who hold a more complex understanding of the organizational constraints, and who find a sense of purpose amidst multiple crises are less negatively impacted by similar levels of surface acting. Therefore, it seems that cognitive reappraisal could contribute to making emotional labor less solely depleting and more like just an element of the professional role.

The above findings support the routine assessment of emotional labor patterns and burnout in psychiatric nursing teams, in order to identify individuals and units where surface acting is particularly intense and cognitive reappraisal resources are limited. Second, cognitive reappraisal is a trainable skill. Programs based on cognitive behavioral principles, resilience training and emotion focused interventions have been shown to reduce burnout and compassion fatigue and to enhance self-compassion in healthcare professionals (38–40). Integrating such training into orientation and continuing education for psychiatric nurses may strengthen their capacity to cope with unavoidable emotional labor. Third, the benefits of emotion regulation training are likely to be limited if organizational conditions remain poor. Current evidence indicates that burnout in nurses is also shaped by staffing levels, exposure to workplace violence, perceived organizational support and the quality of working relationships (25, 26, 29, 30). Individual-level skills training combined with structural enhancements such as safer wards, more equitable workloads, and more transparent systems of recognition would therefore be most promising for the long-term reduction of burnout.

Nonetheless, there are still several limitations. This study adopted a single-center convenience sampling from a psychiatric hospital located in eastern China, causing the findings might not be generalized to other areas or types of hospitals. Emotional labor, emotion regulation, and burnout were all measured by self-report questionnaires, which may potentially induce biases and social desirability effects, especially when the participants are under high work demand. There are several limitations of the present study: the design being cross-sectional does not allow definitive conclusions concerning the direction of causality between surface acting, cognitive reappraisal, and burnout. Future studies should be longitudinal, include multi-center samples, and integrate self-report data with observer ratings, behavioral indicators, and physiological measures. Intervention studies focusing on cognitive reappraisal and other adaptive emotion regulation strategies will also be crucial in assessing whether intentional training can impact the course of burnout in psychiatric nurses.

## 5 Conclusion

In this sample of psychiatric nurses, higher surface acting was associated with higher job burnout, whereas higher cognitive reappraisal was associated with lower burnout. Cognitive reappraisal also showed a small moderating association, with the positive association between surface acting and burnout becoming weaker at higher levels of reappraisal. These findings identify emotion regulation as a potentially relevant correlate of individual differences in burnout but do not establish a causal protective effect. Future longitudinal and intervention studies should evaluate whether cognitive reappraisal training, together with improvements in staffing, workplace safety, workload management, and organizational support, can contribute to burnout prevention.

## Data Availability

The original contributions presented in the study are included in the article/supplementary material. Further inquiries can be directed to the corresponding author.
